# Identification and Characterization of a Novel Gentisate 1,2-Dioxygenase Gene from a Halophilic *Martelella* Strain

**DOI:** 10.1038/srep14307

**Published:** 2015-09-23

**Authors:** Ling Huang, Haiyang Hu, Hongzhi Tang, Yongdi Liu, Ping Xu, Jie Shi, Kuangfei Lin, Qishi Luo, Changzheng Cui

**Affiliations:** 1 State Environmental Protection Key Laboratory of Environmental Risk Assessment and Control on Chemical Process, School of Resources and Environmental Engineering, East China University of Science and Technology, Shanghai 200237, People’s Republic of China; 2 State Key Laboratory of Microbial Metabolism, and School of Life Sciences & Biotechnology, Shanghai Jiao Tong University, Shanghai 200240, People’s Republic of China; 3 Shanghai Engineering Research Center of Contaminated Sites Remediation, Shanghai Institute for Design & Research in Environmental Engineering Co. Ltd., Shanghai 200232, People’s Republic of China

## Abstract

Halophilic *Martelella* strain AD-3, isolated from highly saline petroleum-contaminated soil, can efficiently degrade polycyclic aromatic hydrocarbons (PAHs), such as phenanthrene and anthracene, in 3–5% salinity. Gentisic acid is a key intermediate in the microbial degradation of PAH compounds. However, there is little information on PAH degradation by moderately halophilic bacteria. In this study, a 1,077-bp long gene encoding gentisate 1,2-dioxygenase (GDO) from a halophilic *Martelella* strain AD-3 was cloned, sequenced, and expressed in *Escherichia coli*. The recombinant enzyme GDO was purified and characterized in detail. By using the ^18^O isotope experiment and LC-MS analysis, the sources of the two oxygen atoms added onto maleylpyruvate were identified as H_2_O and O_2_, respectively. The *K*_*m*_ and *k*_*cat*_ values for gentisic acid were determined to be 26.64 μM and 161.29 s^−1^, respectively. In addition, optimal GDO activity was observed at 30 °C, pH 7.0, and at 12% salinity. Site-directed mutagenesis demonstrated the importance of four highly conserved His residues at positions 155, 157, 167, and 169 for enzyme activity. This finding provides new insights into mechanism and variety of gentisate 1,2-dioxygenase for PAH degradation in high saline conditions.

Gentisate (2,5-dihydroxybenzoate) is a key intermediate metabolite in the aerobic degradation of a large number of monocyclic and polycyclic aromatic hydrocarbons (PAHs) by microorganisms. In the gentisate pathway, gentisate is cleaved by gentisate 1,2-dioxygenase (GDO), to form maleylpyruvate^1^. Maleylpyruvate is isomerized to fumarylpyruvate and then hydrolyzed to TCA cycle intermediates, such as fumarate and pyruvate.

Until now, GDOs have been purified and characterized from *Ralstonia (Pseudomonas)* sp. U2^1^, *Klebsiella pneumoniae* M5a1^1^, *Corynebacterium glutamicum* ATCC 13032^2^, *Rhodococcus opacus* R7^3^, *Sphingomonas* sp. RW5^4^, *Xanthobacter polyaromaticivorans*^5^, *Pseudomonas alcaligenes* NCIB 9867 (*xlnE, xbzE*)^6^,^7^, *Haloarcula* sp. D1^8^, *Haloferax* sp. D1227^8^,^9^, *Pseudomonas alcaligenes* NCIMB 9867^10^, and *Silicibacter (Ruegeria) pomeroyi* DSS-3^11^. However, to our knowledge, there is relatively little information about GDO from moderately halophilic bacteria. Comparative studies of the characterization of different gentisate dioxygenases from moderately halophilic bacteria will contribute to the comprehension of the limitations of degrading of PAHs.

*Martelella* sp. strain AD-3, a moderate halophilic bacterium, was isolated from highly saline petroleum-contaminated soil in Shandong province, China^12^. It is highly effective in degrading many PAHs, such as naphthalene, anthracene, and phenanthrene, under broad salinities (0.1–15%) and varying pH (6.0–10.0)^13^. Salicylic acid was also utilized by *Martelella* sp. strain AD-3. At the same time, significantly, high activity of GDO was observed^13^. Furthermore, genes related to degrading gentisic acid has been annotated in the genome sequence of strain AD-3^14^.

In this study, we cloned, expressed, and characterized the gene encoding gentisate 1,2-dioxygenase from *Martelella* AD-3. Moreover, through site-directed mutagenesis, we determined that four His residues in GDO from strain AD-3 are important for its enzymatic activity. Finally, the source of the two oxygen atoms added to the maleylpyruvate was also analyzed.

## Results

### Gene cloning, identification, and amino acid sequence analysis

**Figure 1:**
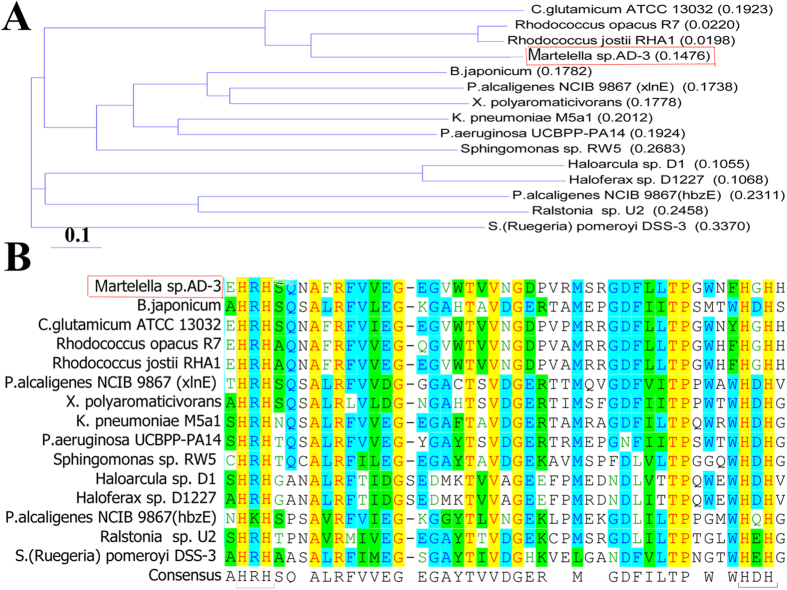
Amino acid sequence analysis results. (**A**) Neighbor-joining tree of the predicted GDO amino acid sequence. (**B**) Excerpts of GDO sequences used for multiple sequence alignment performed by Vector NTI program. Aligned sequences are from halophilic *Martelella* sp. AD-3 (WP_024706076), *Corynebacterium glutamicum* ATCC 13032 (NP_602217.1), *Rhodococcus opacus* R7 (ABH01038.1), *Rhodococcus jostii* RHA1 (ABG93677.1), *Bradyrhizobium japonicum* (NP_766750), *Pseudomonas alcaligenes* NCIB 9867 (xlnE) (AAD49427.1), *Xanthobacter polyaromaticivorans* (BAC98955.1), *Klebsiella pneumoniae* M5a1 (WP_004870415), *Pseudomonas aeruginosa* UCBPP-PA14 (ZP00135722), *Sphingomonas* sp. RW5 (CAA12267.1), Haloarcula sp. D1 (AAQ79814.1), *Haloferax* sp. D1227 (AAQ62856.1), *Pseudomonas alcaligenes* NCIB 9867 (hbzE) (ABD64513.1), *Ralstonia (Pseudomonas)* sp. U2 (AAD12619.1), *Silicibacter (Ruegeria) pomeroyi* DSS-3 (AAV97252.1). The positions of the four highly conserved His residues are indicated in the figures.

The 1,077-bp gentisate 1,2-dioxygenase gene *gdo*, was found by mining the genome sequence of strain AD-3^14^. Amino acid sequence alignment of GDO from strain AD-3 with other related proteins was performed with the Vector NTI program. Compared with other known sequences, the highest identity (70.8%) was found with *Rhodococcus opacus* CIR2 (RnoH, AB186916)^3^, and 60.4% identity with *Corynebacterium glutamicum* ATCC 13032^2^. Amino acid sequence of the AD-3 shared 30.9% and 29.1% sequence identities with two amino acid sequences from *Pseudomonas alcaligenes* NCIB 9867^7^. Compared with other two *Haloferax* strains (*Haloferax* sp. D1 and *Haloferax* sp. D1227)^8^, strain AD-3 had 24.9% and 24.2% identity, respectively. A phylogenetic tree was constructed with GDO proteins from 15 other strains and demonstrated that the protein from strain AD-3 is most closely related to *Rhodococcus opacus* R7^3^ and *Rhodococcus jostii* RHA1^15^ (Fig. 1B).

### Expression of gentisate 1,2-dioxygenase in *E. coli* BL21 (DE3)

**Figure 2:**
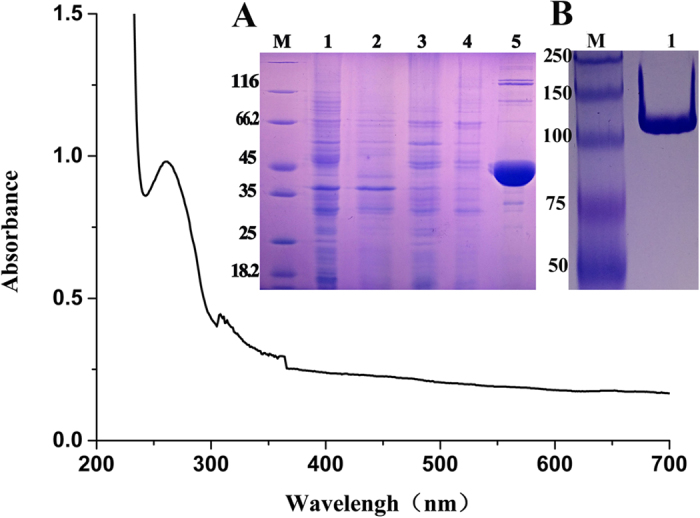
Full wavelength scanning results. (**A**) SDS-PAGE analysis of expressed GDO in BL21 (DE3) on a 12.5% gel. Lane M, protein molecular weight marker (MBI); Lane 1, uninduced cells; Lane 2, cell culture induced with 1 mM IPTG; Lane 3, the supernatant of the induced cells; Lane 4, the precipitate of the induced cells; Lane 5, purified recombinant His_6_-GDO protein; (**B**) NATIVE-PAGE of purified GDO-His_6_. Lane M, protein molecular weight marker (MBI); Lane 1, 5 μl purified GDO-His_6_.

*E. coli* strain BL21(DE3) cells containing the pET28a-*gdo* plasmid, were cultured for 8 h in the presence of IPTG (1 mM) in LB medium at 0, 16, or 30 °C. The most suitable temperature for expression was at 30 °C (Fig. 2A). Protein GDO was purified, and obtained at 12 mg from 1 L LB medium. SDS-PAGE showed the purified protein to be approximately 38 kDa in size (Fig. 2A). Analysis of the eluted fraction by Native-PAGE produced a prominent band of ~120 kDa (Fig. 2B). When stored at −20 °C for 98 h, enzymatic activity was lost.

### Characteristic of the purified GDO

**Figure 3:**
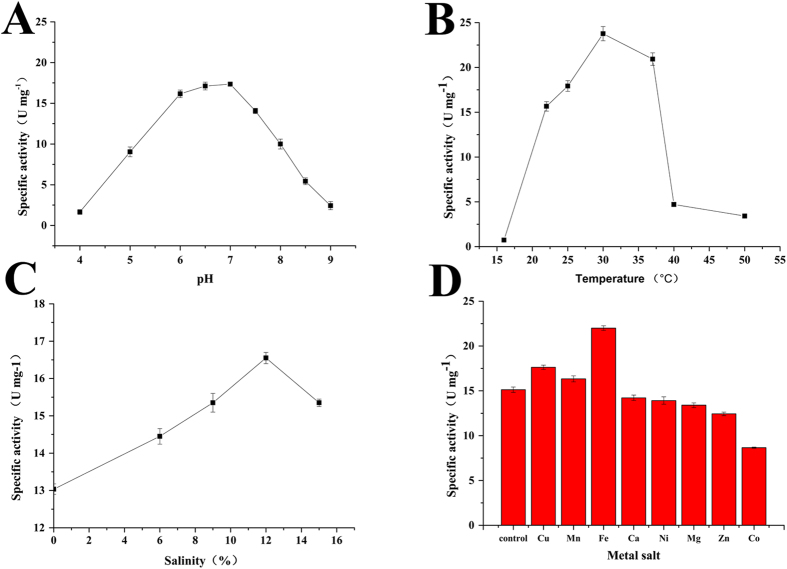
Characterization of GDO. (**A**) pH optimization of GDO. GDO has optimal activity at pH 7.0. (**B**) Temperature sensitivity of GDO enzyme activity; optimal activity was obtained at 30 °C. (**C**) Salinity-dependence of GDO activity; maximum enzyme activity was observed at 12%. (**D**) Effects of metal salts on enzyme activity; control, without metal salt. Ni, Ni^2+^; Co, Co^2+^; Ca, Ca^2+^; Cu, Cu^2+^; Mn, Mn^2+^; Zn, Zn^2+^; Mg, Mg^2+^; K, K^+^; Fe, Fe^2+^.

Effects of pH on GDO activity was measured 5 min after adding GDO to reactions at different pHs. Buffers with pH 4.0–7.0, 7.0–9.0, and 9.0–11.0 were adjusted accordingly with citric acid-sodium citrate, Tris-HCl, and sodium carbonate-sodium bicarbonate buffers, respectively. Enzyme activity of the purified GDO was observed between pH 6.0–8.0 with optimal activity at pH 7.0 (Fig. 3A). Then, effect of temperature on GDO activity was measured at pH 7.0 at temperature ranging from 15–60 °C, maximum enzyme activity was observed at 30 °C (Fig. 3B). Effect of salinity on GDO activity was measured at pH 7.0, 30 °C and salinity ranging from 0 to 16%, optimal activity for the purified GDO was observed at 30 °C, pH 7.0, and salinity at 12% (Fig. 3C). The effects of metal salts at 0.25 mM (Fe^2+^, Cu^2+^, Mn^2+^, Ca^2+^, Ni^2+^, Mg^2+^, Zn^2+^, and Co^2+^) were assessed, indicating that Fe^2+^, Cu^2+^, Mn^2+^ can activate the enzyme, while Ca^2+^, Ni^2+^, Mg^2+^, and Zn^2+^ have no impact on GDO activity. Moreover, the addition of 5 μl Co^2+^ did inhibit enzyme activity to 53% (Fig. 3D).

### Enzyme kinetics of GDO

**Figure 4:**
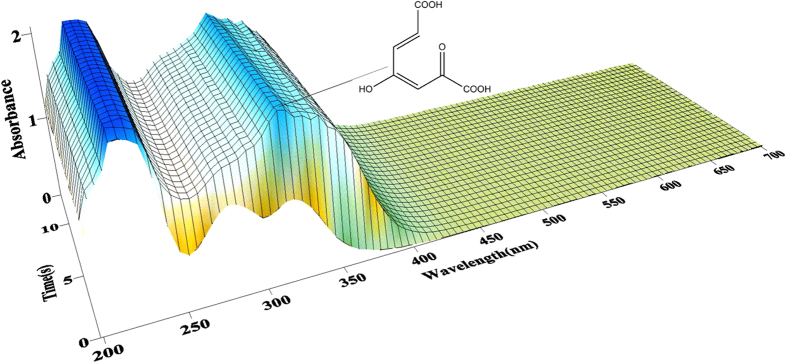
Enzyme kinetics of the purified GDO. A mixture containing 0.46 mM gentisate in 0.1 M phosphate buffer, pH 7.4, was mixed with an equal volume of (2.4 mg/ml) GDO. Absorbance was measured at times ranging from 0 s to 10 s at 4 °C at the UV absorbance spectra, 330 nm.

First, full wavelength scanning of GDO was performed. GDO showed a characteristic absorption peak at 279 nm, which is not the characteristic absorption peak FAD or NADH. Secondly, the result of enzyme kinetics showed that the absorbance at 330 nm steadily increased with time in 1 s, without any transition (Fig. 4). Finally, in order to determine the *K*_*m*_ and *k*_*cat*_ values, GDO activity was assayed at various gentisate concentrations (0–500 mM). The *K*_*m*_ value for gentisate was determined to be 26.64 μM and the *k*_*cat*_ value was 161.29 s^−1^.

### Conserved His residue mutations

Multiple amino acid sequence alignment of GDO sequences from various strains showed the presence of four highly conserved His residues at positions 155, 157, 167, and 169. We designed primers to generate mutants with His to Ala amino acids changes. The resultant mutant plasmids (pET28a-H155A, pET28a-H157A, pET28a-H167A, and pET28a-H169A) were expressed in *E. coli*. The mutant proteins were then purified and the enzyme activity was assayed under the same conditions as for wild type GDO. No enzymatic activity was observed for the four mutant proteins compared to the wild type (Fig. S1).

### ^18^O isotope experiments

**Figure 5:**
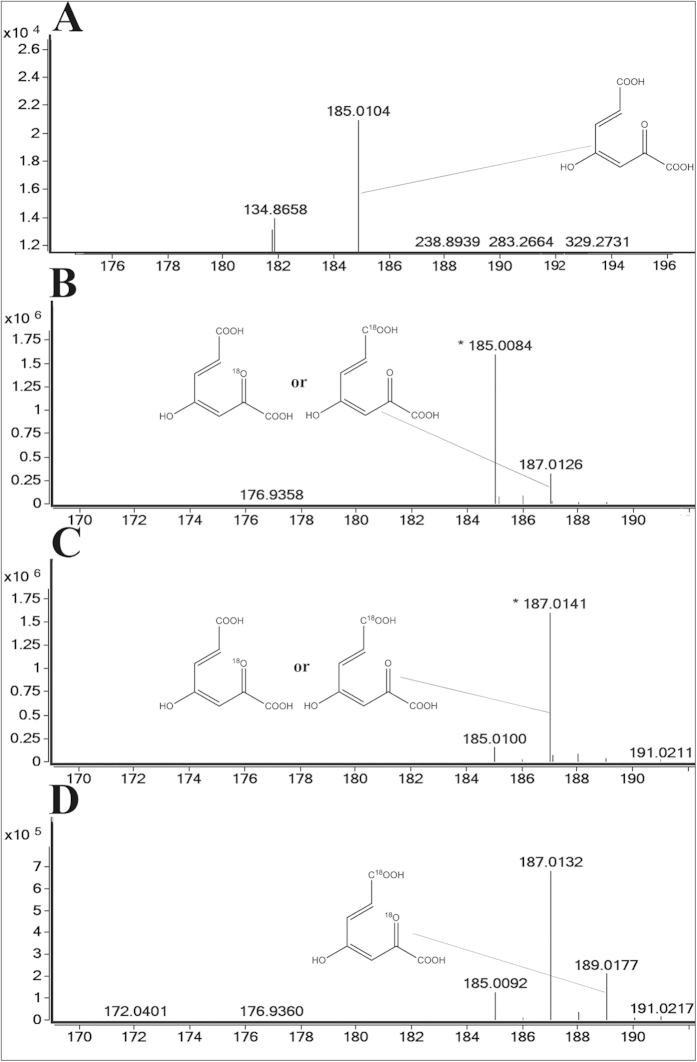
^18^O isotope experiments. (**A**) Control experiment, unlabeled H_2_O was used as a solvent and unlabeled O_2_ was used for the reaction. (**B**) Labeled H_2_^18^O was used as a solvent and unlabeled O_2_ was used for reaction. (**C**) Unlabeled H_2_O was used as a solvent and labeled ^18^O_2_ was used for the reaction. (**D**) Labeled H_2_^18^O was used as a solvent and labeled ^18^O_2_ was used for the reaction.

Two oxygen atoms were added to the degradation product of gentisate, maleylpyruvate. To determine the source of the two oxygen atoms on maleylpyruvate, radio labeled H_2_O or O_2_ were supplied to replace the unlabeled H_2_O or O_2_ in the reaction. The results (Fig. 5) confirmed that the two oxygen atoms added to the maleylpyruvate were from H_2_O and O_2_, one ^18^O labeled maleylpyruvate *m/z* 187.0126 was observed in the H_2_^18^O (Fig. 5B) or ^18^O_2_ (Fig. 5C) experiments and two ^18^O labeled maleylpyruvate *m/z* 189.0177 were observed in the H_2_^18^O and ^18^O_2_ (Fig. 5D) experiment. As a control, the product of the unlabeled H_2_O and O_2_ (Fig. 5A) experiment was analyzed. The labeling experiment results suggest that the two oxygen atoms added to the maleylpyruvate were from H_2_O and O_2_, respectively.

### RT-qPCR analysis

The mRNA levels of the gene *gdo* in strain AD-3 grown in the presence or absence of phenanthrene and gentisic acid were compared using RT-qPCR. The transcript level of gene *gdo* has increased about 6.5 times in the process of degrading phenanthrene and 80.4 times in response to gentisic acid^16^. (Fig. S2).

## Discussion

In recent years, the issue of PAHs pollution has initiated concern among the general public. Specifically, PAHs pollution at high concentrations, in high salinity conditions, and combined pollution^17^ has been of significant concern. Finding microorganisms with the ability to degrade PAHs in high salinity conditions is very important, however, in general, it is difficult for microorganisms to survive under these conditions. A PAH-degrading bacterial strain was previously isolated from highly saline petroleum-contaminated soil^13^ and named as halophilic *Martelella* AD-3. Previous studies indicated that strain AD-3 has a wide spectrum for PAHs substrate degradation though gentisate pathway and a broad range of salinity from 0.1–15%^14^. Therefore, dissecting the specific features of GDO from AD-3 may be helpful to understand the enzymatic mechanisms of GDO.

In this study, gentisate 1,2-dioxygenase from strain AD-3 was purified and characterized. The maximum enzyme activity was observed at 12% salinity. Optimal activity of GDO from *Haloferax* sp. D1227 was reported at 2 M KCl or NaCl (10.4% salinity)^9^. Halophilic proteins function well in high-salt conditions, possessing additional acidic residues (glutamic acid and aspartic acid)^18^. Compared to the amino acid sequence of GDO from *Haloferax* sp. D1227, the sequence from AD-3 contains 16.7% additional acidic residues. This may explain why the higher salinity of 12% was the best condition for its enzymatic activity.

The molecular weight of gentisate 1,2-dioxygenase from strain AD-3 (38 kDa) is similar to GDO from *Ralstonia solanacearum* GMI 1000 (38 kDa)^19^, *Klebsiella pneumoniae* (38 kDa)^1^, *Sphingomonas* sp. RW5 (38.5 kDa)^4^, *Moraxella* sp. VG45 (38 kDa)^1^, *Polaromonas naphthalenivorans* CJ2 (NagI3, 38.7 kDa)^20^, and *E. coli* O157:H7 (38.9 kDa)^21^. A prominent band of ~120 kDa was obtained by Native-PAGE analysis, therefore, we proposed the GDO protein might exist as a trimer protein. The result was the same as the previous reported proteins such as from *Sphingomonas* sp. RW5^4^, *E. coli* O157:H7^21^, *Haloferax* sp. D1227^9^, *Pseudomonas alcaligenes* NCIB 9867^6^. Gentisate 1,2-dioxygenase from strain AD-3 displayed a *K*_*m*_ value (26.64 μM) for gentisate, higher than that from *Sphingomonas* sp. RW5 (15 μM)^4^, *Xanthobacter polyaromaticivorans* 127 W (18.6 ± 1.6 μM)^5^ and *Polaromonas naphthalenivorans* CJ2 (NagI3, 10 μM)^20^. Additionally, GDO from strain AD-3 has an optimal pH of 7.0, similar to that of *Sphingomonas* sp. RW5^4^, and the same optimal temperature 30 °C as *Ralstonia solanacearum* GMI 1000^19^. Above all, the enzyme characteristics of GDO from strain AD-3 are quite similar to those of *Sphingomonas* sp. RW5^4^, with the protein sharing 34.4% amino acid sequence identity^4^.

A previous study reported that Fe^2+^ increased GDO activity by approximately 160% at Fe^2+^ concentrations between 0.05–0.10 mM, for *Pseudomonas alcaligenes* NCIMB 9867^10^. At similar concentrations of Fe^2+^, the activity of GDO from *E. coli* O157:H7 was increased by 115%^21^. For the GDO from strain AD-3, the addition of Fe^2+^ to a final concentration of 0.25 mM only increased enzymatic activity by 34%. The addition of Fe^2+^ was also able to restore part activity of the comparely lost activity GDO from strain AD-3. Cu^2+^, Mn^2+^ can activate GDO from strain AD-3, however the addition of 1 mM Cu^2+^, or 10 mM Mn^2+^ can completely inactivate the GDO from *K. pneumoniae*^21^. The enzyme from *Pseudomonas alcaligenes* NCIB 9867 was inactivated by 5 mM Cu^2+^^7^. The crystal structure of *E. coli*-encoded GDO demonstrates the occupation of three of the possible six iron coordination sites by protein residues, His104, His106, and His145^21^. In this study, in order to assess whether the effect of Fe^2+^ on GDO from strain AD-3 is similar to that of others, we performed site-directed mutagenesis, changing each of the four highly conserved His residues to Ala residues. The results suggest that all four highly conserved His residues are crucial for GDO activity. However, in *Pseudomonas alcaligenes* NCIMB 9867^22^, *Silicibacter (Ruegeria) pomeroyi* DSS-3 (AAV97252.1)^11^, *Klebsiella pneumoniae* M5a1, and *Ralstonia* sp. strain U2^1^, site-directed mutagenesis was performed, changing His residues to Asp residues, based on the results of error-prone PCR mutagenesis of *gdo* from *Pseudomonas alcaligenes* NCIB 9867^6^,^23^. It is possible that GDO could lose its activity when any one of the four His residues was replaced by Asp residues. His and Asp can carry different charges, so the results of the mutagenesis study are not completely convincing. The results in this present study provide further convincing evidence to demonstrate the importance of the four His residues by changing the four His residues to Ala residues.

In this study, we confirmed the source of the two oxygen atoms introduced into maleylpyruvate by ^18^O isotope experiments and subsequent LC-MS analysis. Results suggested that the two oxygen atoms are derived from H_2_O and O_2_. According to the result, we speculated acquiring the oxygen from the O_2_ is ‘easier’ than acquiring it from H_2_O. Because of the traces of ^16^O_2_, the one ^18^O labeled maleylpyruvate *m/z* 187.0126 was observed (Fig. 5D). No isotopic ^18^O form H_2_^18^O was observed in *Pseudomonas*^24^. To *E. coli* O157, H7^21^, *Haloferax* sp. D1227^8^, *K. pneumoniae* M5a1, and *Ralstonia* sp. strain U2^25^, the two oxygen atoms added to maleylpyruvate were reportedly derived only from O_2_. Here, we identified the source of the oxygen atoms by ^18^O isotope labeling experiments, providing a new understanding of the mechanism of gentisate degradation.

In summary, we cloned, sequenced, and characterized a novel gene *gdo* in the degradation of PAHs from *Martelella* strain AD-3. These findings will expand our knowledge of the mechanisms of gentisate 1,2-dioxygenase enzymatic activity.

## Materials and Methods

### Chemicals and media

Gentisic acid (98% purity) was purchased from Sangon Biotech (Shanghai, China). All other reagents and solvents used were analytical grade and the highest purity available. Luria-Bertani (LB) broth (10 g/L tryptone, 5 g/L yeast extract, and 10 g/L NaCl) was used for both culturing and cloning. Solid agar plates were prepared with the addition of 1.5% (w/v) agar to the liquid medium. ^18^O_2_ and H_2_^18^O were purchased from Shanghai Research Institute of Chemical Industry.

### Bacterial strains, plasmids, and growth conditions

*Escherichia coli* DH5α was used as host for recombinant plasmids, and *E. coli* BL21(DE3) (Invitrogen, Carlsbad, CA, USA) was used for expression. The gene *gdo* was amplified from the halophilic *Martelella* strain AD-3. Gene expression plasmids pET28a were obtained from Invitrogen (Invitrogen, Carlsbad, CA, USA). All recombinant cells were grown in LB broth or LB agar plates (15% [w/v]) containing 50 mg/L kanamycin^8^.

### Expression of His_6_-tagged GDO

The gene *gdo* was PCR amplified from the genomic DNA of strain AD-3 with Prime STAR HS DNA polymerase (Takara co. Ltd., Dalia, China)^16^. Primers were designed such that the forward primer contains an *Nde*I site and the reverse primer contains a *Hind*III site. The primer sequences were as follows, forward, 5′-GCCGCATATGAACATGATGATGCCTGAAGA-3′ and reverse, 5′-ATTAAGCTTTCATGCGTCTGCGTCTTCGACC-3′ (*Nde*I and *Hind*III recognition sites are underlined). PCR amplification was performed with 100-μl reaction mixtures containing 50 pmol of each primer, 10 μl of a deoxynucleoside triphosphate mixture, 250 ng of template DNA, and 50 μl of 2 × Prime Star buffer. PCR was carried out by using the following program, 5 min at 94 °C and then 30 cycles of 30 s at 94 °C, 30 s at 58 °C, and 2 min 30 s at 72 °C. The PCR products were purified, digested with *Nde*I and *Hind*III, and then ligated into pET28a (Novagen Corp., Germany), which had been double digested with the same restriction enzymes. The pET28a-*gdo* plasmid was transformed into *E. coli* BL21(DE3). DNA restriction enzymes and T4 DNA ligase were purchased from New England Biolabs^26^. *E. coli* BL21(DE3) cells containing pET28a-*gdo*, were cultured at 37 °C in LB medium containing 50 μg/ml kanamycin to an OD_600_ of 0.6–0.8. Then, after adding IPTG to a final concentration of 1 mM, the cultures incubated for 8 hours at 16 °C or 20 °C or 30 °C to express GDO. The cells were harvested and resuspended in binding buffer (25 mM Tris-HCl, 300 mM NaCl, 20 mM imidazole, pH 8.0) at an OD_600_ of 30. Cells were then lysed by sonication on ice, and centrifuged at 10,000 × *g* for 20 min to separate the soluble cell lysate from the insoluble membrane and protein aggregates^5^.

### Purification of GDO

The supernatant was filtered through a 0.22 μm filter and the resultant filtrate was applied to a 5-ml column of Ni-NTA agarose (GE, Healthcare, Little Chalfont, UK), which had been equilibrated with the binding buffer. After a wash with 30 ml of washing buffer (25 mM Tris-HCl, 300 mM NaCl, 70 mM imidazole, pH 8.0), His_6_-tagged GDO was eluted from the column with elution buffer (25 mM Tris-HCl, 300 mM NaCl, 200 mM imidazole, pH 8.0). All purification steps were carried out at 4 °C.

### Enzyme assays and protein determination

Gentisate 1,2-dioxygenase activity was spectrophotometrically assayed at 330 nm by measuring maleylpyruvate formation^5^. Activity was assayed in 1 ml of reaction mixture containing 0.46 mM gentisate in 0.1 M phosphate buffer, pH 7.4, at 23 °C with a UV-2550 spectrophotometer (Shimadzu, Kyoto, Japan). A molar extinction coefficient of 10.8 × 10^3^ M/cm was used to calculate specific activity^20^. One enzyme unit was defined as the amount of enzyme that produces 1 mmol of maleylpyruvate per min at 23 °C^7^. Enzyme activity was assayed 5 min after adding the enzyme to the reaction mixture.

### Rapid reaction kinetics

The enzyme kinetics was measured with an SFM 4000 stopped flow apparatus (BioLogic, France). Constant temperature was maintained in a JULABO model F21 temperature-controlled bath (Julabo, Seelbach, Germany). Spectral scans were recorded with a TIDAS S 300 K diode array detector (J&M Analytik AG, Germany)^27^. Experimental parameters were set as follows, enzyme/substrate solutions, 1/1; UV absorbance spectra, 330 nm; injection rate, 20 μl/s; detection times, 10 ms to 10 s; temperature, 4 °C. Additionally, the enzyme concentration was no lower than 5 mg/ml and the gentisic acid were the same as in the enzyme assays.

### Site-directed mutagenesis

Site-directed mutagenesis was performed using a recombinant PCR method. Primers are listed in Table 1. Mutant genes were subcloned into a pET28a vector between the *Nde*I and *Hind*III restriction sites, separately. All mutant strains were analyzed by sequencing to confirm disruption of the target gene^28^.

### DNA and amino acid sequence analysis

Amino acid sequences of GDO from other strains were obtained from GenBank. All homology searches were carried out on the NCBI BLAST server (http,//www.ncbi.nlm.nih.gov/BLAST) with the nucleotide BLAST and protein BLAST. These obtained GDO sequences were then compared with the sequence from AD-3. Conserved binding domain searches were performed using Vector NTI DNA analytical software (version 11.0).

### ^18^O isotope experiments

To ascertain the origin of the two oxygen atoms added onto maleylpyruvate, H_2_^18^O was added to the solvent for the gentisate reaction or ^18^O_2_ was added to the anaerobic environment. Then, the assay mixtures of dried GDO powder and gentisic acid dissolved in H_2_^18^O were incubated at 30 °C for 20 min. Another assay mixture dissolved in H_2_^16^O was incubated at the same temperature for 20 min, injected with ^18^O_2_ at the beginning. As a control, another mixture dissolved in H_2_^16^O was prepared for reacting in the ^16^O_2_ atmosphere. After termination of the reaction by adding 2 volumes of ethanol, samples were analyzed using liquid chromatography-electrospray ionization-mass spectrometry (LC-ESI-MS) (samples were prepared by filtration)^28^, the negative mode of ESI**-**MS analysis was used to monitor the products.

### Liquid chromatography-mass spectrometry (LC-MS) analysis

Identification of GDO degradation products was carried out by LC-MS (Agilent, Pale Alto, CA, USA) using ESI in the negative ion mode. Substrate solutions were prepared by adding two volumes of ethanol to precipitate the enzyme and filtered through a 0.22-μm Millipore filter 80 min after adding 0 μl or 1 μl GDO enzyme. Then, the samples were automatically injected (5 μl) into the high performance liquid chromatography system. Separation was achieved by ion-pair chromatography on a Luna C18 5 μm column (4.6 × 150 mm, Keystone Scientific, Bellefonte, PA), at 30 °C. Eluent A was deionized H_2_O (18 MΩ/cm, 0.05% formic acid [v/v]), and eluent B was acetonitrile (99.9%). The working conditions were 90% eluent A and eluent B 10% [v/v] at an injection rate of 0.4 ml/min. The UV absorbance spectra were obtained at 330 nm, on-line^16^.

### RT-qPCR analysis

Total RNA was isolated from strain AD-3, which was cultured in glycerol medium or induced medium with the presence of phenanthrene or gentisic acid, using an RNeasy mini kit (TIANGEN, China)^16^. Reverse transcription PCR (RT-PCR) was performed using a Prime Script one-step RT-PCR kit (Takara, Japan). Quantitative PCR reactions were carried out using 0.2 ml qPCR tubes (Bio-Rad) and a Chromo 4 real-time PCR thermocycler (Bio-Rad). Reaction was performed by using 20-μl reaction mixtures containing 9 μl PerfeCTa SYBR Green Fast Mix (TIANGEN, China), 0.4 μl of each primer, 10 ng DNA sample and DNase free water to a final volume of 20 μl. The standard curve for each pair of primer was constructed with a tenfold dilution of genomic DNA from strain AD-3^26^. The 16S rRNA gene was used as control. For gene *gdo*, the thermocycler program used for qPCR was as follow: 95 °C for 3 min, 34 cycles of 20 s at 95 °C, 10 s at 63.2 °C/50.9 °C, and 15 s at 68 °C, then 95.0 °C for 1 min, 55.0 °C 1 min and 5 s, stored at 95 min. The primers for RT-qPCR were 5′-AAGAGGTAAGTGGAATTG-3′ and 5′-CAGTAATGGACCAGTAAG-3′ for 16S rRNA, 5′-ATGATGATGCCTGAAGACA-3′ and 5′-GAGCGGATTGAGGTGATT-3′ for the gene *gdo*. The threshold cycle (Ct) values for gene *gdo* from three different conditions, were normalized to the reference gene, 16S rRNA gene. The relative expression level was calculated by using the 2 ^–ΔΔCt^ method, where ΔΔCt = (Ct target—Ct 16S rRNA) _INDUCTION_—(Ct target—Ct 16S rRNA) _CONTROL_^16^.

## Additional Information

**How to cite this article**: Huang, L. *et al.* Identification and Characterization of a Novel Gentisate 1,2-Dioxygenase Gene from a Halophilic *Martelella* Strain. *Sci. Rep.* **5**, 14307; doi: 10.1038/srep14307 (2015).

## Supplementary Material

Supplementary Information

## Figures and Tables

**Table 1:** Primers for site-directed mutagenesis.

**Points**	**Sequence(5′–3′)**
H_155–F_	AGCCCGTCATTCCCAGAATGCCTTCCGTTT
H_155–R_	CATTCTGGGAATGACGGGCTTCGGGCGCCGTTTC
H_157–F_	ACACCGTGCCTCCCAGAATGCCTTCCGTTT
H_157–R_	CATTCTGGGAGGCACGGTGTTCGGGCGCCG
H_167–F_	CTTCGCCGGCCACCACAACGAAACCGACCA
H_167–R_	CGTTGTGGTGGCCGGCGAAGTTCCAGCCGGG
H_169–F_	GCCCACAACGAAACCGACCAGCCCATGGCC
H_169–R_	GGTTTCGTTGTGGGCGCCGTGGAAGTTCCA
