# Supplementary material for: Identification and Characterization of a Novel Gentisate 1,2-Dioxygenase Gene from a Halophilic Martelella Strain
Source: Sci Rep. 2015 Sep 23;5:14307. doi: 10.1038/srep14307 (PMC4585797; doi:10.1038/srep14307)
Supplement: Supplementary Information [file srep14307-s1.pdf]

Supplementary Information

Identification and Characterization of a Novel Gentisate 1,2-Dioxygenase Gene from a Halophilic *Martelella* Strain

Ling Huang[1], Haiyang Hu[2], Hongzhi Tang[2#], Yongdi Liu[1], Ping Xu[2], Jie Shi[1], Kuangfei Lin[1], Qishi Luo[3] and Changzheng Cui[1#]

[1] State Environmental Protection Key Laboratory of Environmental Risk Assessment and Control on Chemical Process, School of Resources and Environmental Engineering, East China University of Science and Technology, Shanghai 200237, China

[2] State Key Laboratory of Microbial Metabolism, and School of Life Sciences & Biotechnology, Shanghai Jiao Tong University, Shanghai 200240, People's Republic of China

[3] Shanghai Engineering Research Center of Contaminated Sites Remediation, Shanghai Institute for Design & Research in Environmental Engineering Co. Ltd., Shanghai 200232, People's Republic of China

[#]Address correspondence to, Hongzhi Tang, School of Life Sciences and Biotechnology, Shanghai Jiao Tong University, Shanghai 200240, PR China. Tel, +86-21-34206647; Fax, +86-21-34206647-801; E-mail, tanghongzhi@sjtu.edu.cn

[#]Address correspondence to, Changzheng Cui, State Environmental Protection Key Laboratory of Environmental Risk Assessment and Control on Chemical Process, School of Resources and Environmental Engineering, East China University of Science and Technology, Shanghai 200237, China. E-mail, cuichangzheng@ecust.edu.cn

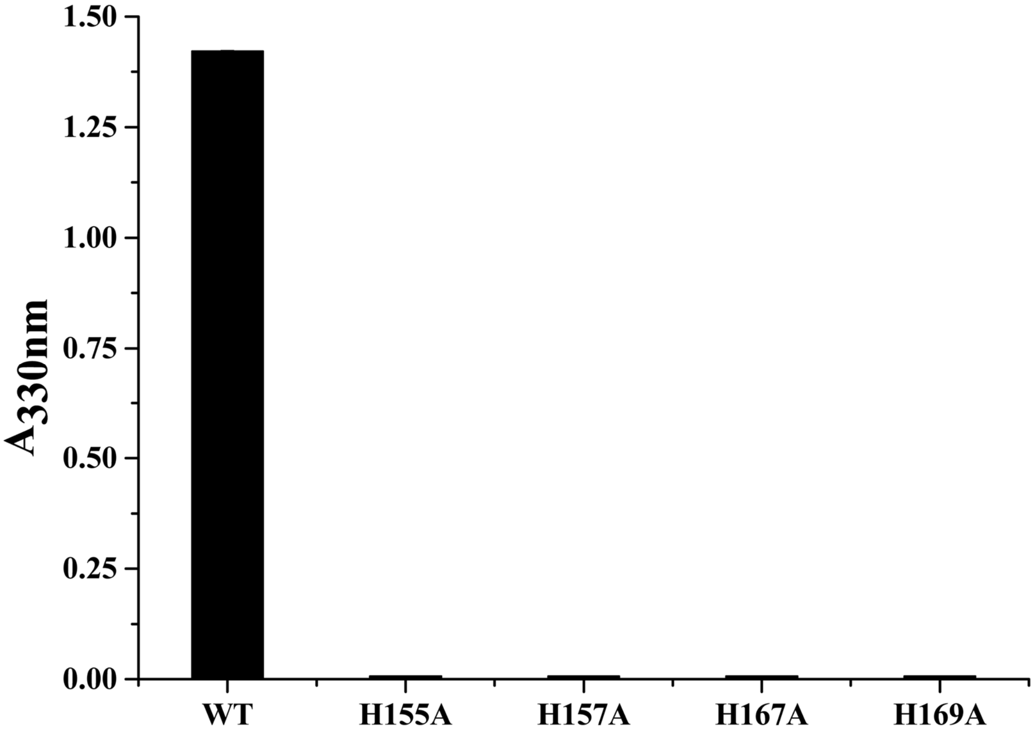

Fig. S1. Enzyme assay for the four mutant proteins. All mixtures of the purified proteins and substrates were incubated at 30°C. Control, wild type protein; H155A, H157A, H167A, and H169A mutant proteins. Reaction consisted of 1 μl wild type or mutant protein in the reaction mixture (1 ml total volume, containing 0.46 mM gentisate in 0.1 M phosphate buffer, pH 7.4).

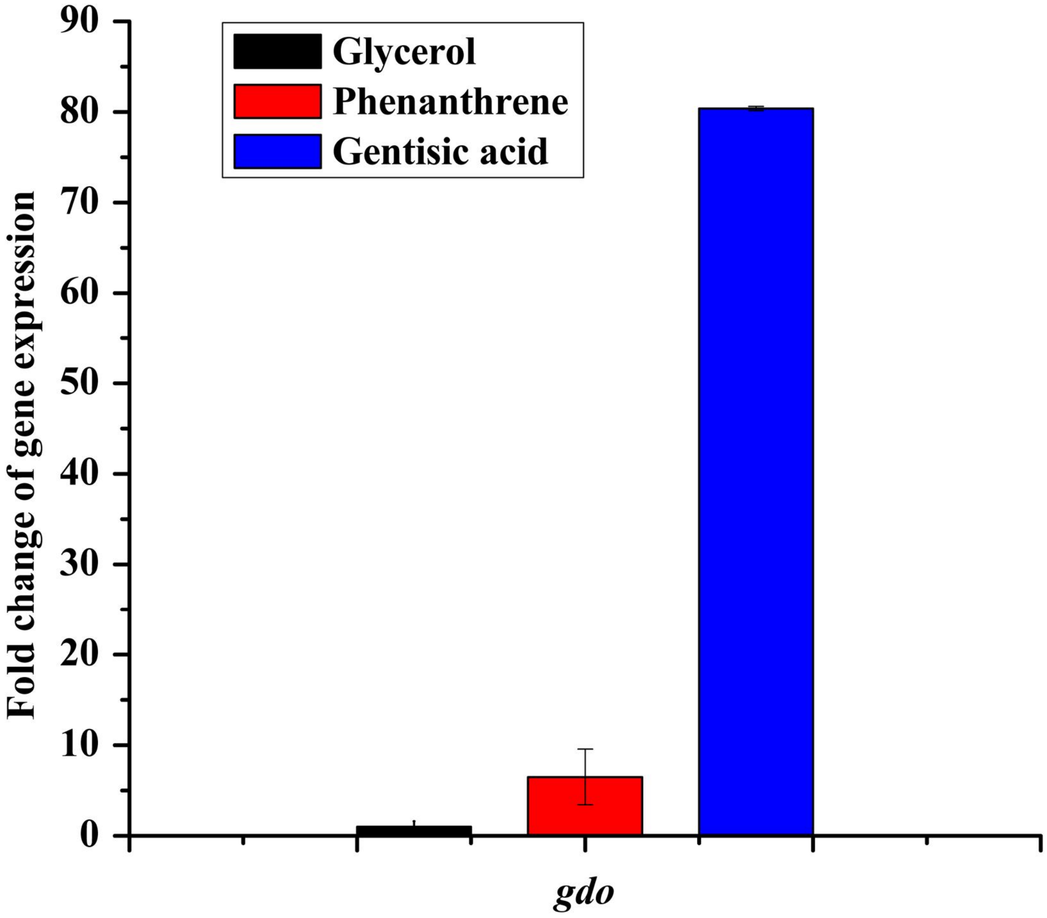

Fig. S2. RT-qPCR analysis of the expression level of gene *gdo*. The plot shows expression level of gene *gdo* in different medium: black, glycerol medium; red, phenanthrene medium; blue, gentisic acid medium. Bars show the average error.
